# Population genetic structure of domain I of apical membrane antigen-1 in *Plasmodium falciparum* isolates from Hazara division of Pakistan

**DOI:** 10.1186/s12936-018-2539-3

**Published:** 2018-10-26

**Authors:** Sahib Gul Afridi, Muhammad Irfan, Habib Ahmad, Muneeba Aslam, Mehwish Nawaz, Muhammad Ilyas, Asifullah Khan

**Affiliations:** 1 Department of Biochemistry, Abdul Wali Khan University Mardan, Mardan, Khyber Pakhtunkhwa 23200 Pakistan; 2 Center for Human Genetics, Hazara University, Mansehra, 21310 Pakistan; 3 Center for OMIC Studies, Islamia College University, Peshawar, 25000 Pakistan

**Keywords:** *P. falciparum*, Population genetics, *pfama1*, Pakistan

## Abstract

**Background:**

The *Plasmodium falciparum* apical membrane antigen-1 (PfAMA1) is considered as an ideal vaccine candidate for malaria control due to its high level of immunogenicity and essential role in parasite survival. Among the three domains of PfAMA1 protein, hyper-variable region (HVR) of domain I is the most immunogenic. The present study was conducted to evaluate the extent of genetic diversity across HVR domain I of the *pfama1* gene in *P. falciparum* isolates from Hazara division of Pakistan.

**Methods:**

The HVR domain I of the *pfama1* was amplified and sequenced from 20 *P. falciparum* positive cases from Hazara division of Pakistan. The sequences were analysed in context of global population data of *P. falciparum* from nine malaria endemic countries. The DNA sequence reads quality assessment, reads assembling, sequences alignment/phylogenetic and population genetic analyses were performed using Staden, Lasergene v. 7.1, MEGA7 and DnaSP v.5 software packages respectively.

**Results:**

Total 14 mutations were found in Pakistani isolates with 12 parsimony informative sites. During comparison with global isolates, a novel non-synonymous mutation (Y240F) was found specifically in a single Pakistani sample with 5% frequency. The less number of mutations, haplotypes, recombination and low pairwise nucleotide differences revealed tightly linked uniform genetic structure with low genetic diversity at HVR domain I of *pfama1* among *P. falciparum* isolates from Hazara region of Pakistan. This uniform genetic structure may be shaped across Pakistani *P. falciparum* isolates by bottleneck or natural selection events.

**Conclusion:**

The Pakistani *P. falciparum* isolates were found to maintain a distinct genetic pattern at HVR *pfama1* with some extent of genetic relationship with geographically close Myanmar and Indian samples. However, the exact pattern of gene flow and demographic events may infer from whole genome sequence data with large sample size of *P. falciparum* collected from broad area of Pakistan.

**Electronic supplementary material:**

The online version of this article (10.1186/s12936-018-2539-3) contains supplementary material, which is available to authorized users.

## Background

Malaria is a vector-borne and infectious disease which is caused by a peripheral blood protozoan parasite of the genus *Plasmodium* [1], including five different species: *Plasmodium falciparum, Plasmodium vivax, Plasmodium knowlesi, Plasmodium malariae* and *Plasmodium ovale* [2]. The *P. falciparum* species accounts for the majority of the medical cases leading to lethal malaria [3, 4]. Malaria is prevalent in subtropical and tropical countries particularly Asia and Africa. In spite of advances in knowledge, the malaria disease continues to cause significant health care burden worldwide [5]. Malaria continues to have a great impact on the adults and children health all over the world. In 2016, it caused 429,000 deaths and 212 million clinical cases [5]. Malaria is considered widespread in 104 countries and territories worldwide [5]. The control and elimination for malaria is challenging due to spread of *Plasmodium* resistance to anti-malarial drugs along with insecticide-resistant *Anopheles* mosquitoes. Effective vaccine development is urgently required for better combat of malaria infection. The circumsporozoite protein (CSP), merozoite surface protein-1 (MSP-1), apical membrane antigen-1 (AMA1), and thrombospondin related anonymous protein (TRAP) are reported as vaccine candidate proteins for *P. falciparum* [6]. However, the genetic polymorphisms in these parasite proteins create hurdles in development of effective vaccines [7]. These polymorphisms change the critical epitopes expression and eventually reduce or cause complete loss of vaccine efficacy [8]. Therefore, extensive evaluation of genetic variants in these vaccine candidate antigenic proteins in *P. falciparum* populations from malaria endemic regions is primarily important for an effective and enduring vaccine development.

The AMA1 is integral membrane protein expressed in the merozoite and sporozoite stages of *P. falciparum* life cycle. This protein is considered to play a crucial role in invasion of erythrocytes and hepatocytes by *Plasmodium* [9]. The AMA1 immunization elicits antibodies production and effectively inhibits the erythrocyte invasion by the parasite [10], making AMA1 a leading vaccine candidate [11]. The AMA1 protein is comprised of three domains, and domain I exhibits high sequence polymorphism and is shown to be a key target of anti-AMA-1 protective antibodies [12]. The hyper-variable region (HVR) of domain I is highly immunogenic and natural immune responses have been reported against this domain [13]. Several studies have reported the higher rate of non-synonymous (dN) mutations at this domain due to strong diversifying selection [12, 14].

Pakistan is endemic for malaria and 60% of its population is living in malaria-endemic regions. An average 50,000 deaths occur each year in Pakistan due to malarial infection. Malaria is mostly caused by *P. vivax and P. falciparum* all around the Pakistan [15]. The malaria transmission remains throughout the year in Pakistan with maximum intensity from July to November [16]. The genetic structure of *pfama1* gene has been reported from different malaria endemic regions of the world [12, 17, 18]. However, no report is available so far about the genetic architecture of *pfama1* gene from Pakistan. The present study investigated the extent of genetic polymorphism at HVR domain I of the *pfama1* among *P. falciparum* isolates collected from low malaria endemic Hazara division of Pakistan (i.e., termed as Pakistani Hazara, PKH).

## Methods

### Study area and samples collection

**Fig. 1:**
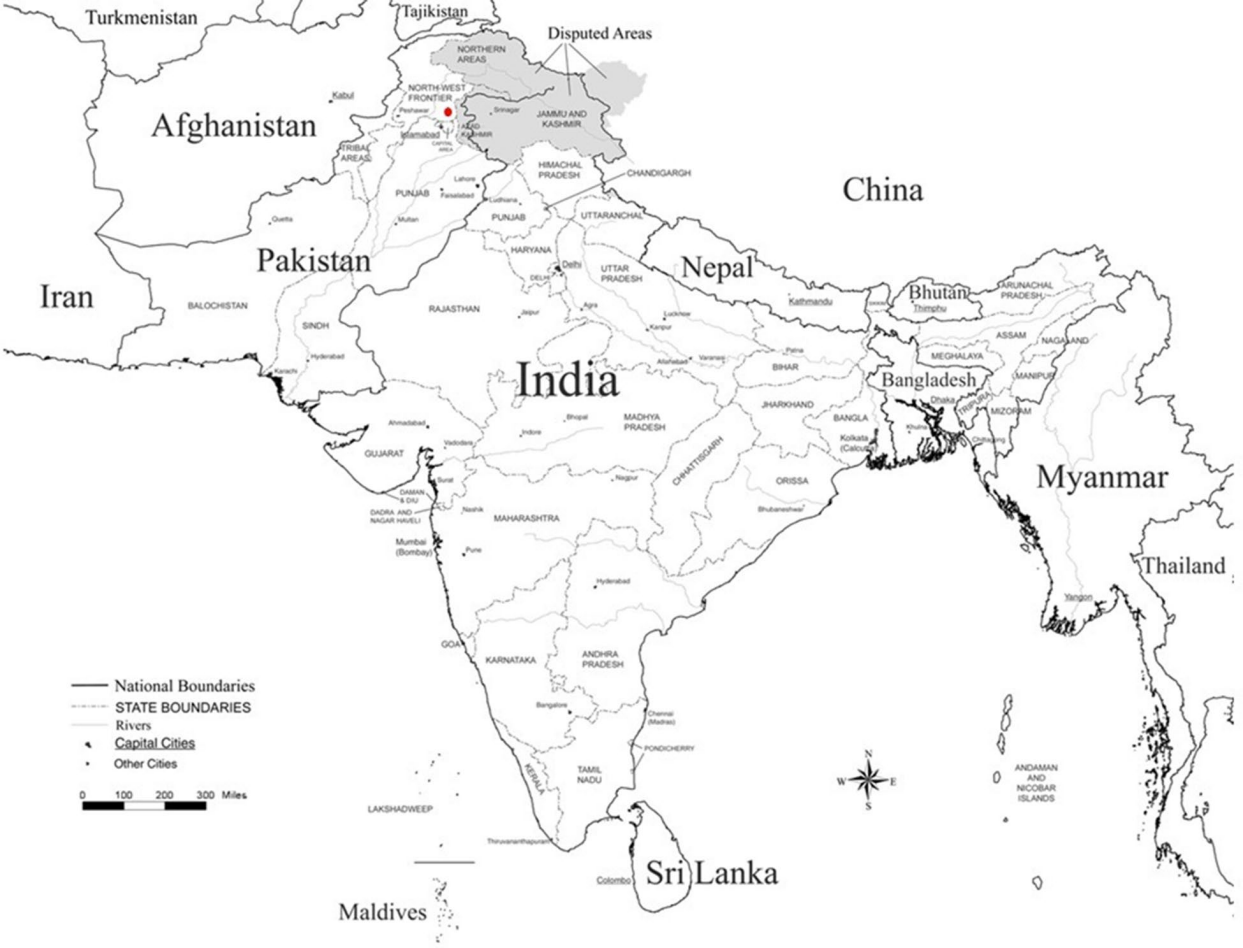
Map of Pakistan, India, Myanmar and Thailand. The red dot shows the study region (i.e. Hazara division of Pakistan)

The study is carried out from Hazara division situated immediately south of the main Himalayas range in the North-Eastern part of the Khyber Pakhtunkhwa province of Pakistan (Fig. 1). Total 1961 malaria positive patients were confirmed by microscopic analysis from major hospitals of three districts (Abbottabad, Haripur and Mansehra) of Hazara division during malaria season (July–November, 2016). During data collection, 0.37 malaria cases per 1000 individuals of Hazara population were observed. According to the World Health Organization, the malaria cases > 1 per 1000 individuals of a population may consider as high transmission malaria region. The age of both male and female patients ranged from 11 to 57 years (average 28.3 years) and the infection tendency of male and female patients was found 55 and 45%, respectively in target area. Among total malaria positive cases, only 31 patients (1.6%) were diagnosed *P. falciparum* positive by Giemsa-stained thick smears. The blood samples (2 ml) of 20 *P. falciparum* positive patients were collected for DNA extraction.

The institutional ethical guidelines were followed during blood samples collection and informed consent was obtained from all the study participants. The DNA was purified from whole blood samples using Thermo Scientific Gene JET Genomic DNA Purification Kit.

### PCR amplification and DNA sequencing analysis of HVR domain I *pfama1*

The DNA samples were subjected to *pfama1* gene HVR locus amplification through nested PCR technique using specific primers and PCR conditions reported by Garg et al. [12]. The PCR amplified products were examined through 2% agarose gel electrophoresis. The 500 bp DNA band was excised from agarose gel and purified by QIA quick Gel Extraction kit (Qiagen, Hilden, Germany) following the manufacturer’s instructions. A unidirectional Sanger-based sequencing of 500 bp purified PCR product was carried out using ABI3130 Analyzer (Applied Biosystem, USA). The cycle sequencing reactions were performed using Big Dye Terminator kit (Applied Biosystems Inc, USA) with supplier recommended conditions. The sequencing of each PCR product was performed in triplicate from single end and mutations were verified from the high-quality trace data. The sequence reads trace quality check was performed using the Staden Package [19]. The gap4 in Staden package assigns confidence values to base call which is a numerical estimate of base calling accuracy. The confidence values are stored in experiment files and copied into gap4 during data entry. All the sequence reads were evaluated on this basis and low quality reads were discarded or trimmed. Mutations were confirmed on the basis of high confidence values from triplicate (3×) sequence reads. The sequence reads assembling was performed using Lasergene package version 7.1 (DNASTAR Inc. USA). The low-quality sequences were trimmed and total 417 bp good quality sequence data was generated for each sample.

### Nucleotide sequence polymorphism analysis

The nucleotide sequence polymorphism analysis of 20 Pakistani Hazara (PKH) samples was conducted. The numbers of segregating sites (S), haplotypes (H), haplotype diversity (Hd), nucleotide diversity (π), Tajima’s D, McDonald-Kreitman test, pairwise nucleotide differences within a population (K), recombination events (Rm) and Linkage disequilibrium (LD) were calculated using DnaSP v. 5 [20].

### Genetic diversity analysis of HVR *pfama1* among global *Plasmodium falciparum* isolates

The HVR *pfama1* sequences of *P. falciparum* populations from Ghana, Tanzania, Papua New Guinea (PNG), India, Philippines, Thailand, Vanuatu and Solomon Islands were retrieved from GenBank NCBI and comparative genetic analyses were performed (Additional file 1). The samples size (n = 20) and nucleotide sequence length (i.e., 417 bp) were kept same for different parasite populations to perform unbiased statistical analysis. The allele frequencies were calculated manually on the basis of percentage prevalence of observed allele in total number of samples. The statistical analyses including genetic diversity, sequences alignment and phylogenetic analysis were conducted using DnaSP v 5 [20] and MEGA7 programs [21].

### Epitope prediction

The Immune Epitope Database (IEDB) server [22] was used for PfAMA1 MHC class I and II epitopes prediction. The SMM (Stabilized matrix method) and SMM-align (netMHCII-1.1) methods were followed for human MHC class I and class II epitopes predictions respectively. The top binders with the cut-off value of IC_50_ < 100 nM were shortlisted to check the potential impact of the discovered nsSNPs with respect to alteration of the core sequences of lead epitopes.

### Sequences submission to GenBank NCBI

The final draft of high quality DNA sequences of PKH samples were deposited to GenBank under Accession Number; MH028212-MH028193 and PopSet: 1368343752.

## Results

### Sequence polymorphism of PKH in HVR *pfama1* sequences

**Fig. 2:**
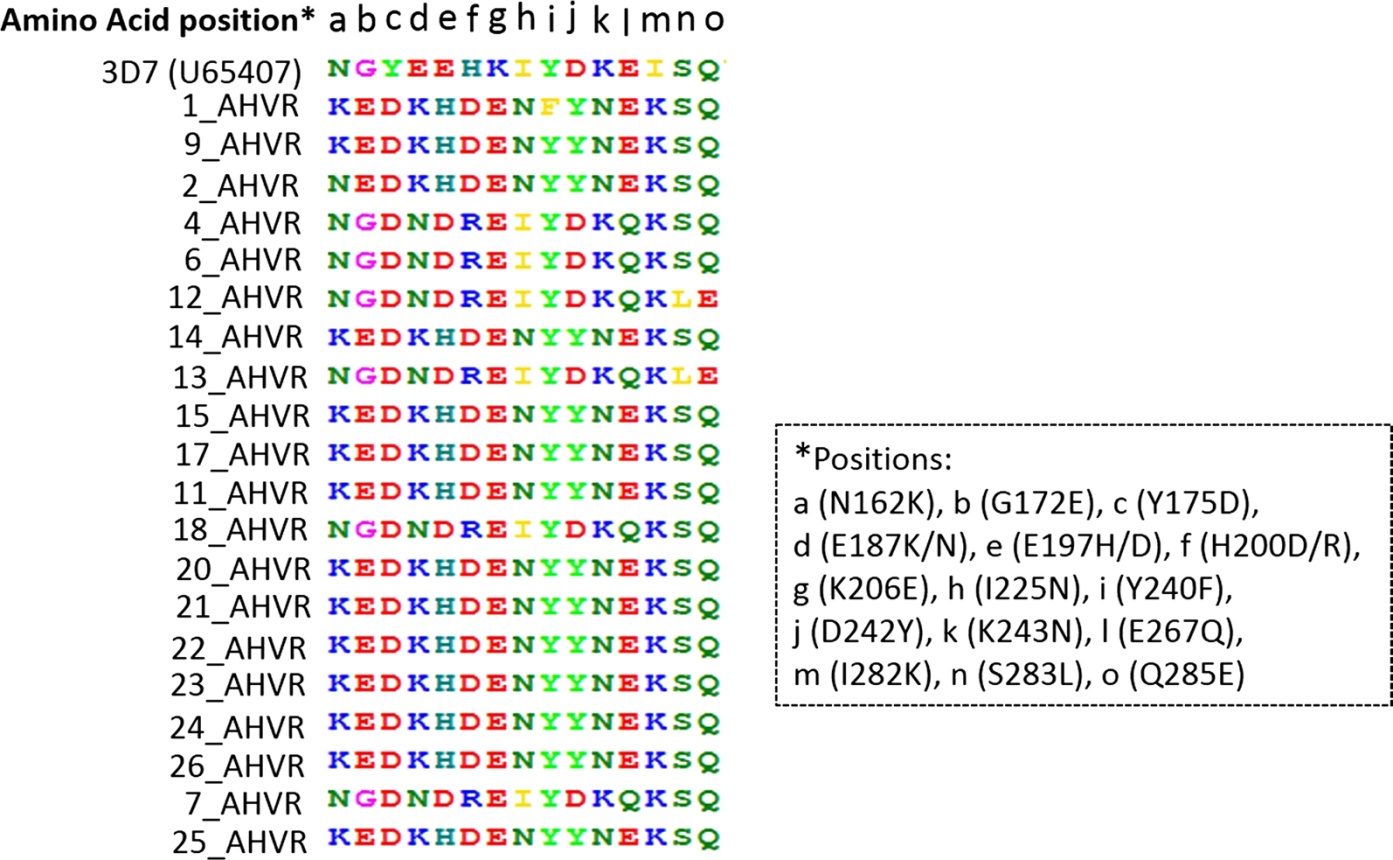
The polymorphic amino acid positions of *P. falciparum* isolates HVR *pfama1* sequences from Hazara division of Pakistan. The 3D7 (GenBank Accession No.: U65407) is reference *P. falciparum* sequence

The partial nucleotide sequences of 417 bp obtained for 20 PKH *P. falciparum* samples, spanning nucleotide positions 464–880 of reference 3D7 *pfama1* sequence (Accession Number: U65407). The nucleotide sequence analysis of these sequences compared to reference revealed total 20 single nucleotide polymorphisms (SNPs), including 2 synonymous SNPs and 18 non-synonymous SNPs (nSNPs) in PKH *P. falciparum* isolates. These SNPs resulted in amino acid change at 15 positions in PKH samples sequences (Fig. 2). Three amino acids changes (i.e. tyrosine (Y) 175 to aspartic acid (D), lysine (K) 206 to glutamic acid (E), and isoleucine (I) 282 to lysine (K) were found fixed in all PKH samples sequences.

### Population genetic analyses of PKH samples in context of global isolates

**Fig. 3:**
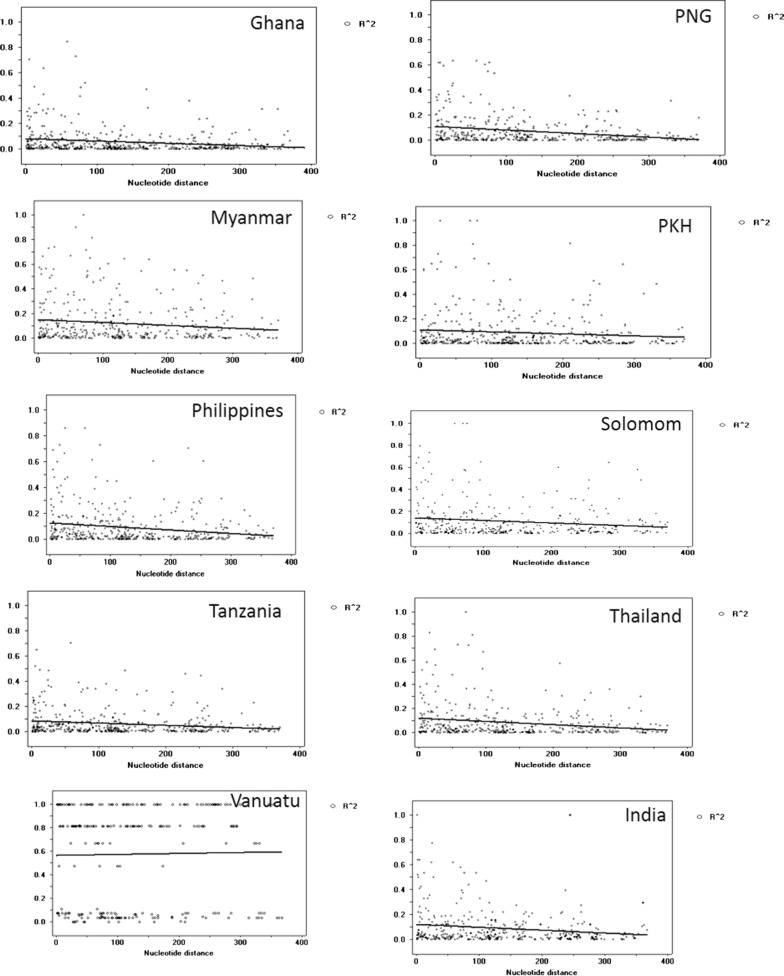
Linkage disequilibrium (LD) plot in global *pfama1* including Pakistani isolates. The LD index (R^2^) at Y-axis was plotted against nucleotide distance across HVR *pfama1* at X-axis using a two-tailed Fisher’s exact test. PKH is Pakistani Hazara, PNG is Papua New Guinea

The nucleotide diversity of HVR loci at *pfama1* of PKH *P. falciparum* isolates were analysed in context of worldwide samples deposited in GenBank (see, “Methods” section). Overall a distinct genetic structure of PKH samples were found in terms of less number of mutations, and lower values for haplotype diversity (Hd), pairwise nucleotide differences within population (K), and pairwise nucleotide diversity (π) compared to samples from other regions (Table 1). Likewise, low recombination event approaching zero and high linkage disequilibrium (LD) values were found for PKH samples compared to samples from other geographical regions (Table 2, Fig. 3). This revealed tight linkage at HVR loci for PKH samples. The LD index (R^2^) was found to decreases with increasing distance for target region as like most of other global samples (Fig. 3). The highest recombination events were found for African samples from Ghana and Tanzania across HVR loci as reported in previous study based on complete *pfama1* gene sequences analysis [18]. The haplotype (H), Hd, K and π values calculated for PKH samples were observed close to samples from Myanmar (Table 1). In pairwise Hd and K analysis, least population-wise differences were found among Myanmar, India and PKH isolates (Table 3). This reveals close genetic relationship among geographically nearby Myanmar, India, Thailand and PKH *P. falciparum* isolates.

**Table 1:** The estimates of DNA sequence polymorphism and tests of neutrality at *pfama1* among PKH *P. falciparum* isolates along with global samples

Isolates	Segregating sites (S)	Singleton variable sites	Parsimony informative sites	Total no. of mutations	K	H	Hd ± SD	π	Tajima’s D	Fu and Li’s D	Fu and Li’s F
Ghana	35	7	28	39	12.04211	16	0.979 ± 0.021	0.02888	0.37976 (P > 0.10)	0.37631 (P > 0.10)	0.43962 (P > 0.10)
PNG	28	2	26	31	11.495	13	0.963 ± 0.023	0.02757	1.23920 (P > 0.10)	1.29980 (P > 0.05)	1.49238 (P > 0.05)
India	33	7	26	37	11.053	19	0.9947 ± 0.00032	0.02651	0.23721 (P > 0.10)	− 0.00337 (P > 0.10)	0.07966 (P > 0.10)
Myanmar	25	7	18	26	6.326	9	0.653 ± 0.122	0.01517	− 0.53086 (P > 0.10)	− 0.06044 (P > 0.10)	− 0.23238 (P > 0.10)
PKH	35	2	12	14	5.395	5	0.616 ± 0.106	0.01294	1.34376 (P > 0.10)	0.77839 (P > 0.10)	1.09423 (P > 0.10)
Philippines	29	4	25	30	11.053	9	0.911 ± 0.032	0.02651	1.20358 (P > 0.10)	0.72573 (P > 0.10)	1.01009 (P > 0.10)
Solomon	24	0	24	25	10.263	4	0.742 ± 0.056	0.02461	1.76659 (0.10 > P > 0.05)	1.62535 (0.10 > P < 0.02)	1.93964 (0.10 > P < 0.02)
Tanzania	35	6	29	39	11.979	16	0.963 ± 0.033	0.02873	0.35690 (P > 0.10)	0.52264 (P > 0.10)	0.55126 (P > 0.10)
Thailand	26	0	26	27	10.021	10	0.916 ± 0.040	0.02403	1.23278 (P > 0.10)	1.43415 (P < 0.05)	1.59960 (0.10 > P > 0.05)
Vanuatu	23	3	20	23	9.874	4	0.616 ± 0.067	0.02368	2.01117 (P < 0.05)	0.90227 (P > 0.10)	1.43009 (P > 0.10)

*PKH* Pakistani Hazara, *PNG* Papua New Guinea

**Table 2:** Comparison of recombination events at HVR *pfama1* among different pathogen populations

Isolates	Ra	Rm
Ghana	0.3293	10
PNG	0.1918	8
India	0.3293	8
Myanmar	0.0000	3
PKH	0.0000	0
Philippines	0.0697	6
Solomon	0.0221	3
Tanzania	0.2812	10
Thailand	0.0743	6
Vanuatu	0.0012	3

*Ra* recombination parameter between adjacent sites, *Rm* minimum number of recombination events between adjacent sites, *PKH* Pakistani Hazara, *PNG* Papua New Guinea

**Table 3:** Estimation of pairwise haplotype diversity (Hd) and population average nucleotide differences (K) at HVR *pfama1* among different *P. falciparum* population

Population	Ghana	PNG	Myanmar	PKH	Phil	Solomon	Tanzania	Thailand	Vanuatu	India
Ghana	0	11.829	10.304	9.354	11.641	11.588	11.977	11.108	11.599	11.517
PNG	0.986	0	10.013	9.671	11.310	11.196	11.933	11.087	11.126	11.315
Myanmar	0.910	0.873	0	7.282	10.022	10.153	10.149	8.714	8.713	9.531
PKH	0.901	0.897	0.822	0	9.344	9.342	9.515	8.235	9.214	8.415
Phil	0.971	0.946	0.858	0.885	0	11.065	11.827	10.971	10.705	11.099
Solomon	0.928	0.895	0.845	0.844	0.885	0	11.777	10.694	10.932	11.272
Tanzania	0.982	0.981	0.905	0.897	0.965	0.921	0	11.196	11.605	11.458
Thailand	0.974	0.958	0.858	0.886	0.942	0.881	0.971	0	10.397	10.710
Vanuatu	0.9013	0.863	0.8115	0.8128	0.8051	0.7718	0.8974	0.8628	0	10.904
India	0.9910	0.9897	0.9141	0.8731	0.9731	0.9359	0.9897	0.9782	0.9038	0

The Hd values given in the lower left quadrant and K in the upper right quadrant

*PKH* Pakistani Hazara, *PNG* Papua New Guinea, *Phil* Philippines

### Non-synonymous SNPs pattern of PKH in context of worldwide samples

Among non-synonymous (nsSNPs) the glycine (G) 172 to Glutamic acid (E), E187K(lysine)/N(Asparagine) and the E197Q(Glutamine)/G/D/H(Histidine) are reported as the high level of polymorphic amino acid changes in global *pfama1* sequences [18]. These nsSNPs were found in PKH sequences as well. The E187K/N nsSNP was found as K187N substitution in PKH samples with major (i.e., 70%) K amino acid. Likewise at 197 position, H to D amino acid substitution was observed in PKH samples with predominant “H” amino acid at 70% frequency (Fig. 2). The Q285E was observed at low frequency (i.e. 10%) as like samples from other populations reported previously [18]. The serine (S) 283 to leucine (L) nsSNP was found with 10% frequency in PKH samples as like samples from Tanzania, Ghana, Solomon and PNG reported by Kang et al. [18]. In Myanmar and Thailand, the frequency of this nsSNP was found comparatively high, i.e. close to 40% [18]. The I (Isoleucine) 282 to K nsSNP was found fixed in PKH samples with “K” amino acid only. The high frequency of this SNP has also been reported in samples from Philippines, Ghana, and Tanzania. However the I282K frequency is reported comparatively low (i.e. < 50%) in Vanuatu, Solomon, Thailand and Myanmar [18]. The E267Q substitution was found in PKH samples with a frequency of 30%, which is more or less similar to other population samples. Only in Myanmar the frequency of E267Q substitution is reported considerably low, i.e., close to 10% [18]. The I225N, D242Y (tyrosine) and K243N substitutions were found in PKH samples with more or less same frequency as reported for other populations [18]. In some populations D242A substitution is reported, however in PKH samples no “A” substitution was found as a consequence of nsSNP at 242 residue. The K206E substitution was found fixed in PKH samples and high substitution frequency of this SNP is reported for samples from Tanzania, Ghana, Solomon, PNG, Philippines and Thailand. However, its frequency is reported comparatively low in Myanmar (i.e. < 50%) [18]. The H200D/L/R nsSNP is reported as tetra-morphic amino acid change in different continental populations, but in case of PKH samples, only D to R substitution was found at this position with predominant “D” amino acid (i.e. 70%). Likewise, the E197Q/G/D/R/H/V is reported as penta-morphic amino acid change in Myanmar and other geographical samples, however in PKH samples, it was found as H to D substitution only with predominant “H” amino acid with 70% frequency. The E187K/N nsSNP was found as K to N substitution in PKH samples with 70% frequency of “K”. The Y175D substitution was found as fixed nsSNP in PKH. This nsSNP is reported as fixed change in Myanmar *pfama1* sequences as well. High frequency of Y175D substitution is reported in Africa, Oceania and East Asian samples [18]. The G172E amino acid substitution was found in PKH samples with 70% frequency of amino acid “E”. High frequency of this nsSNP is also reported in samples from Tanzania, Ghana, Thailand and Myanmar. However, in Vanuatu this amino acid substitution frequency is reported quite low, i.e., approximately 10% [18]. The N162K substitution was also found high in PKH samples with “K” amino acid frequency of 70%. This amino acid substitution frequency is reported low, i.e. close to 30% in PNG, Thailand, Myanmar and African samples from Ghana and Tanzania [18]. All the above mentioned nsSNPs identified in PKH isolates are reported in *P. falciparum* isolates from India. However, the detail substitution frequency of these nsSNPs is not mentioned for Indian isolates [12]. A novel Y240F substitution was found specifically in a single PKH sample (i.e., 5% frequency) during comparative sequence analysis with 459 sequences from GenBank for globally available samples. Besides, this nsSNP has not been reported in any previously published literature reports about *pfama1*gene analysis.

### Phylogenetic analysis

**Fig. 4:**
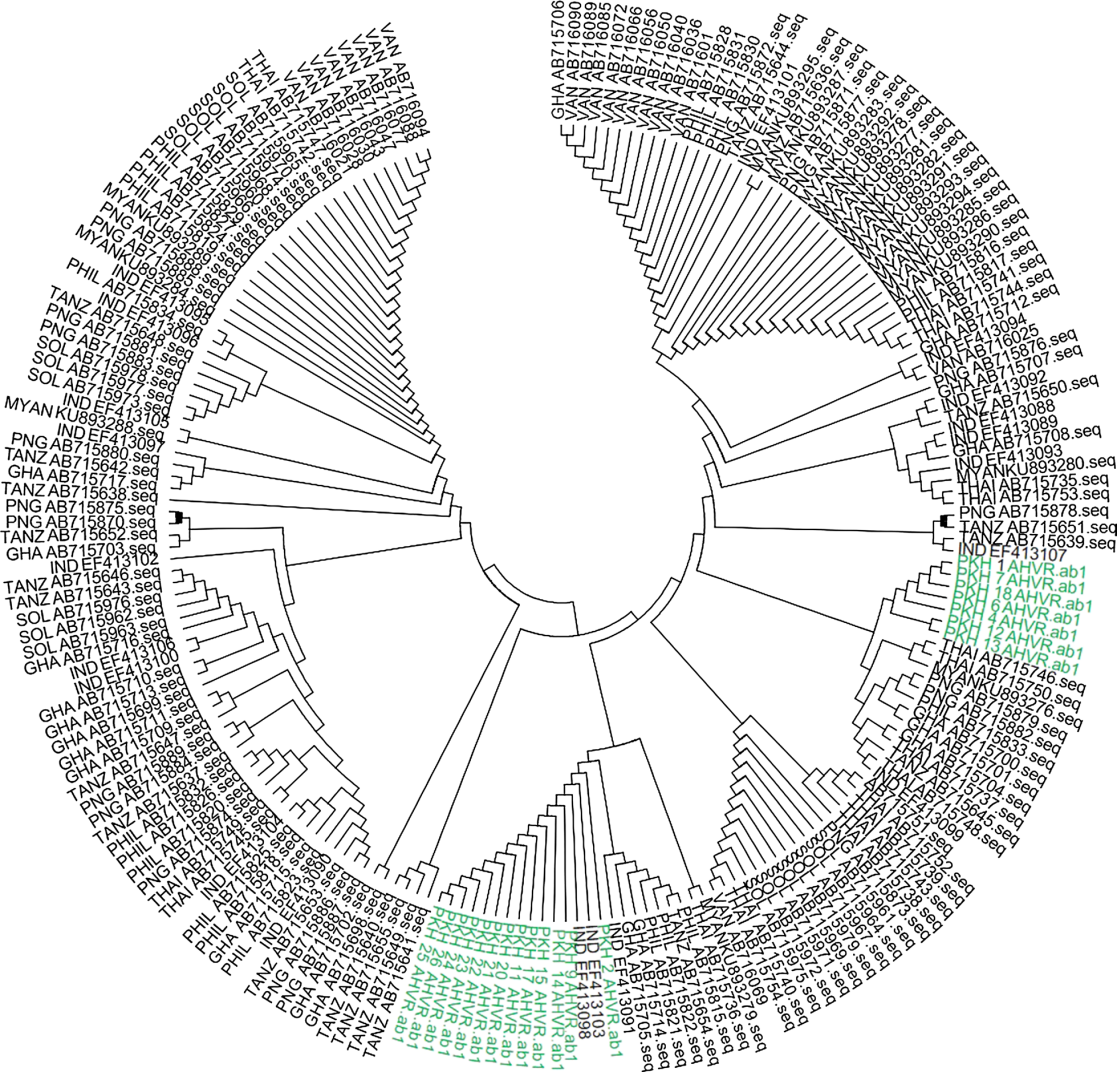
The neighbour joining phylogenetic tree constructed on the basis of HVR *pfama1* sequences from PKH and global samples. The green color indicates PKH samples. Samples codes started with GHA is Ghana, TANZ is Tanzania, PHIL is Philippines, PNG is Papua New Guinea, SOL is Solomon, MYAN is Myanmar, IND is India, VAN is Vanuatu, and THAI is Thailand

The neighbour joining (NJ) method based phylogenetic analysis of PKH samples were carried out along with global *pfama1* HVR sequences. Mostly the geographically distinct isolates were found to develop separate clade, however, somewhere different geographical isolates shared the same tree clade (Fig. 4). The PKH samples were found to develop two separate clusters in phylogenetic tree. However, in one major cluster the PKH samples were found to share clade with Indian, Thailand and Ghana samples (Fig. 4).

## Discussion

It becomes essential to investigate the antigenic repertoires of the *P. falciparum* populations from different malaria endemic regions. The genetic structure of Pakistani *P. falciparum* isolates was analysed from malaria endemic Hazara division in context of global populations based on HVR domain I of *pfama1* gene. The natural selection and recombination events are previously reported as main driving forces that bring genetic diversity across *pfama1* in global populations [12, 17, 18]. This adaptive evolutionary pattern of *pfama1* is reported in association with host immune pressure, which eventually contributes in host immune escape [18]. The analyses of PKH samples *pfama1* sequences along with worldwide *P. falciparum* isolates with equal samples size, result in identification of lowest haplotype number, haplotype diversity, recombination events and mutations for PKH samples as compared to global isolates (Tables 1, 2, 3). This revealed somehow the uniform genetic structure with low genetic diversity across HVR loci of *pfama1* in PKH samples as compared to *P. falciparum* global isolates. The lower genetic diversity of PKH samples might be due to low endemicity of *P. falciparum* genotypes in Hazara division or might arouse due to bottleneck or natural selection. Though the Tajima’s D test result was not found statistically significant for PKH samples (Table 1), however the positive values somehow indicate the tendency of natural selection and deviation from neutral evolution as like previous studies [23–25]. The exact demographic event responsible for low genetic diversity of PKH isolates might be confirmed from whole genome sequence data with large samples size to be collected from broader regions of Pakistan.

The overall pattern of amino acid substitution in PfAMA1 for PKH samples were found similar to global sequences. No tri or tetra morphic amino acid changes were observed in PKH samples. However, the amino acid substitution frequencies observed for PKH samples were found different at several loci compare to samples from other malarial endemic regions. A novel Y240F amino acid substitution was observed at low frequency only in a single PKH sample. As limited number of samples is analysed in this study from a confined region of Pakistan, therefore, these amino acid changes and their frequency may not be statistically significant to represent the actual amino acid substitution spectrum of *pfama1* gene across overall Pakistani region.

Most of the observed amino acid substitutions identified in our study were predicted to affect the PfAMA1 lead epitopes binding with human MHC class I and class II. Among these the H200D/R, K206E, K243N, I282K, S283L and Q285E were found to alter the PfAMA1 lead epitopes (IC_50_ cut off < 100 nM) cores residues and predicted to influence the epitopes binding with human host HLA-DRB1*01:01 (MHC-II). Likewise, the nsSNPs N162K, E197H/D, G172E, Y175D, I225N, I282K, S283L and Q285E were found to alter the lead epitope core sequences and suspected to influence its binding with human HLA-A*01:01 (MHC-I) allele. A novel nsSNP (i.e. Y240F) identified for a PKH sample only was not found to influence the top epitopes (i.e. IC_50_ cutoff < 100 nM) core sequences.

The genetic structure of HVR *pfama1* gene of PKH isolates was found unique in comparison to global *P. falciparum* isolates sequences deposited at GenBank. During the pairwise Hd and nucleotide differences (K) analyses, the PKH isolates were found close to Myanmar samples. This might be due to close geography of PKH and Myanmar populations of the parasite. The phylogenetic tree analysis was found incongruent to this finding and the PKH and Myanmar isolates were found to develop far away independent tree clades (Fig. 4). The *P. falciparum* samples from India were actually collected as separate populations from different geographical regions of India as reported [12]. However, the separate population detail of these isolates is not given for DNA sequences data deposited at GenBank. Twenty of these Indian *P. falciparum* HVR sequences were selected to infer their genetic relationship with PKH samples. The high values of H, Hd and K for Indian samples in our analysis reflects their sub-population genetic heterogeneity. As a result, the Indian samples were found scattered throughout the phylogenetic tree and failed to develop a distinct tree clade (Fig. 4). Though Indian samples showed least difference with PKH isolates as compared to any other population in pairwise Hd and K analysis (Table 3), which reflects their close genetic affinity, however the sub-population genetic structure of Indian samples may bias such pairwise analyses. A close genetic relationship of Indian samples with PKH was also supported somehow by phylogenetic analysis as well and Indian samples were found to share a tree clade with PKH samples sequences.

## Conclusion

Distinct genetic structure of *pfama1* gene from *P. falciparum* isolates is reported from different malaria endemic regions. Therefore, a country-wide analysis is required to understand the pattern of genetic diversity across this important vaccine candidate gene. The PKH *P. falciparum* isolates were found to hold distinct genetic structure compared to global isolates. Overall the PKH samples exhibit uniform genetic structure with low inter-population diversity in comparison to global isolates. The population genetic analysis indicates the genetic relationship of PKH samples with geographical nearby *P*. *falciparum* isolates from Myanmar and India. Additional studies with large samples size may further validate the exact pattern of gene flow of *P*. *falciparum* isolates from Pakistani corridors into Central and South Asian regions. Likewise, the whole genome sequence data analysis will require to identify the exact demographic event responsible for low genetic diversity across HVR *pfama1* in *P*. *falciparum* isolates from Pakistan.

## Additional file

**Additional file 1:** The NCBI accession numbers of *pfama1* sequences of *P. falciparum* global populations used during comparative sequence analysis with PKH samples. PNG is Papua New Guinea.
