# Supplementary material for: Population genetic structure of domain I of apical membrane antigen-1 in Plasmodium falciparum isolates from Hazara division of Pakistan
Source: Malar J. 2018 Oct 26;17:389. doi: 10.1186/s12936-018-2539-3 (PMC6203999; doi:10.1186/s12936-018-2539-3)
Supplement: Supplementary file 1 — Additional file 1: The NCBI accession numbers of pfama1 sequences of P. falciparum global populations used during comparative sequence analysis with PKH samples. PNG is Papua New Guinea. [file 12936_2018_2539_MOESM1_ESM.docx]

| **Sample** | **Accession Numbers** |
| --- | --- |
| **Ghana** | AB715698, AB715700, AB715706, AB715701, AB715707, AB715702, AB715703, AB715709, AB715705, AB715712, AB715708, AB715699, AB715704, AB715713, AB715711, AB715715, AB715716, AB715717, AB715710, AB715714. |
| **PNG** | AB715870, AB715875, AB715871, AB715872, AB715877, AB715874, AB715878, AB715876, AB715879, AB715880, AB715881, AB715882, AB715883, AB715884, AB715873, AB715885, AB715887, AB715888, AB715886, AB715889. |
| **Myanmar** | KU893288, KU893284, KU893276, KU893289, KU893279, KU893280, KU893295, KU893283, KU893292, KU893278, KU893287, KU893277, KU893281, KU893282, KU893291, KU893293, KU893294, KU893285, KU893286, KU893290. |
| **Philippines** | AB715820, AB715816, AB715821, AB715817, AB715818, AB715822, AB715819, AB715823, AB715824, AB715825, AB715826, AB715827, AB715830, AB715831, AB715832, AB715833, AB715828, AB715829, AB715829, AB715815. |
| **Solomon** | AB715961, AB715963, AB715962, AB715964, AB715978, AB715966, AB715967, AB715979, AB715969, AB715960, AB715976, AB715971, AB715972, AB715973, AB715977, AB715968, AB715974, AB715970, AB715975, AB715965. |
| **Thailand** | AB715735, AB715740, AB715736, AB715741, AB715738, AB715742, AB715743, AB715745, AB715746, AB715747, AB715748, AB715737, AB715749, AB715739, AB715751, AB715750, AB715752, AB715744, AB715754, AB715753. |
| **Tanzania** | AB715638, AB715642, AB715639, AB715644, AB715643, AB715641, AB715645, AB715646, AB715636, AB715637, AB715649, AB715648, AB715650, AB715651, AB715652, AB715653, AB715654, AB715655, AB715640, AB715647. |
| **Vanuatu** | AB716010, AB716015, AB716016, AB716012, AB716017, AB716018, AB716019, AB716021, AB716011, AB716013, AB716022, AB716023, AB716014, AB716024, AB716025, AB716020, AB716027, AB716028, AB716029, AB716026. |
| **India** | EF413103, EF413098, EF413090, EF413104, EF413100, EF413106, EF413102, EF413097, EF413096, EF413095, EF413101, EF413094, EF413088, EF413089, EF413093, EF413107, EF413099, EF413091, EF413103, EF413098 |

**Table S1**: The NCBI accession numbers of *pfama1* sequences of *P. falciparum* global populations used during comparative sequence analysis with PKH samples. PNG is Papua New Guinea.
